# The ACT-i-Pass study protocol: How does free access to recreation opportunities impact children’s physical activity levels?

**DOI:** 10.1186/s12889-015-2637-x

**Published:** 2015-12-23

**Authors:** Jason A. Gilliland, Andrew F. Clark, Patricia Tucker, Harry Prapavessis, William Avison, Piotr Wilk

**Affiliations:** Human Environments Analysis Lab, University of Western Ontario, Social Science Centre, London, N6A 5C2 ON Canada; Children’s Health Research Institute, 800 Commissioners Rd. E., London, N6C 2V5 ON Canada; Lawson Health Research Institute, 268 Grosvenor St, London, N6A 4V2 ON Canada; Department of Geography, University of Western Ontario, Social Science Centre, London, N6A 5C2 ON Canada; Department of Paediatrics, University of Western Ontario, 800 Commissioners Rd. E., London, N6C 2R6 ON Canada; School of Health Studies, University of Western Ontario, Arthur and Sonia Labatt Health Sciences Building, London, N6A 5B9 ON Canada; School of Occupational Therapy, University of Western Ontario, Elborn College, London, N6G 1H1 ON Canada; School of Kinesiology, University of Western Ontario, Thames Hall, London, N6A 5B9 ON Canada; Department of Sociology, University of Western Ontario, Social Science Centre, London, N6A 5C2 ON Canada; Department of Epidemiology & Biostatistics, University of Western Ontario, Kresge Bldg, London, N6A 5C1 ON Canada

## Abstract

**Background:**

Physical activity during childhood is associated with a multitude of physical, behavioural, and psychological health benefits. Identification of effective population level strategies for increasing children’s physical activity levels is critical for improving the overall health of Canadians. The overall objective of this study is to assess how a naturally-occurring, community-level intervention which offers Grade 5 children in London, Canada a free access pass to physical activity opportunities (facilities and programs) for an entire school year can lead to increased physical activity among recipients.

**Methods/Design:**

This study adopts a longitudinal cohort study design to assess the effectiveness of improving children’s access to physical activity opportunities for increasing their physical activity levels. To meet our overall objective we have three aims: (1) to assess whether the provision of free access increases children’s physical activity levels during and after the intervention compared to a control group; (2) to assess how and why child-specific trajectories of physical activity (between-children differences in level of physical activity measured across time) in the intervention group differ according to children’s individual and household characteristics; and (3) to explore additional factors that are unaccounted for in the theoretical model to gain a further understanding of why the free access intervention had varying effects on changing physical activity levels. We will be addressing these aims using a mixed methods approach, including: a series of youth surveys conducted before, during, immediately after, and 4-months after the intervention; parent surveys before, during, and post-intervention; real-time tracking of the access pass use during the intervention; and focus groups at the conclusion of the intervention. Data compiled from the youth surveys will provide a subjective measure of physical activity to be used as our outcome measure to address our primary aims.

**Discussion:**

The results of this study can inform policy- and decision-makers about the sub-groups of the population that benefitted the most (or least) from the intervention to provide more specific information on how to develop and target future interventions to have a greater impact on the physical activity levels and overall health of children.

## Background

Declining levels of physical activity (PA) have been identified as a major cause of rising obesity rates among Canadian children [1, 2]. Physical activity during childhood is associated with a multitude of physical, behavioural, and psychological health benefits [3–8]. Unfortunately, only 5 % of Canadian children aged 5–17 years meet the nation’s recommended guidelines of 60 min of moderate-to-vigorous intensity PA on most days of the week [9]. Identification of effective strategies for increasing children’s PA at the population level is critical for improving the overall health of Canadians. The purpose of this study is to assess how a naturally-occurring community-level intervention offering free access to PA opportunities (facilities and programs) can lead to increased PA among children.

### Factors associated with children’s physical activity

Research indicates that children’s PA is influenced by multiple factors at different levels: individual (e.g., age, sex, immigrant status, Aboriginal status, socio-economic status); interpersonal (e.g., parental and peer support); and community (e.g., density of recreation facilities, availability of PA programs) [10–12]. Little attention has been given to how these factors explain variation in PA change among different subgroups of children exposed to the same intervention [13]. This type of understanding would enable researchers to identify subgroups of children that may benefit most from an intervention, thus being able to tailor future interventions for effective PA change.

With regard to individual-level socio-demographic factors, a negative association has been identified between PA and age, with PA declines being steepest for female adolescents [14, 15]. In general, boys tend to be more active than girls in Canada [16–19], but study results have been mixed [20]. Research in Canada and the United States has also found that PA levels are lower among new and recent immigrants [21–27]. Although most previous studies have focused on adults, one Canadian study found only 32 % of new immigrants participate in organized PA once a week compared to 55 % for non-immigrants [22]. Studies using the Canadian Community Health Survey have reported that no significant differences exist in the prevalence of PA among Aboriginal versus non-Aboriginal youth [28, 29], but obese Aboriginal youth are significantly less likely to participate in PA than their non-obese peers [29], and the prevalence of obesity is nearly twice as high for Aboriginal versus non-Aboriginal youth (15.8 % vs 8.0 %) [28]. Examination of the socio-economic status-PA relationship in children and youth has also produced equivocal study findings [11, 30]; however, some socio-economic status indicators (e.g., parent income and education levels) have been shown to have a strong association with children’s participation in structured PA [17, 31–33].

Children’s interpersonal networks can also influence their PA behaviour. Several studies suggest that parents can have a positive influence on their children’s PA through supportive actions, such as: providing transportation to PA opportunities, watching children participate in PA, and actively playing with children [34–37]. Indeed, parents’ PA behaviours can also influence children’s PA participation, although conflicting evidence makes it difficult to know the true extent of this relationship [12]. Some studies have found a significant relationship between parent and child PA [38–41]; however, others have failed to find a significant relationship [40, 42–44]. In general, studies indicate that children are more physically active when they have supportive friends and peers [45–49], while negative peer interaction can significantly decrease PA [50–53]. Additionally, overweight children who have a positive social network that is supportive of PA tend to also experience a benefit in PA levels, compared to comparable children without peer support [48, 54].

More recent research has focussed on community or neighbourhood effects on PA levels among children [20]. Studies by our team [20] and others [55] have revealed that poor access to PA opportunities is associated with lower PA levels among children. While being proximate to PA opportunities is important in shaping the ability to carry out healthy behaviours [56].

### Intervention studies

Reviews of the effectiveness of PA interventions have demonstrated very limited efficacy for changing children’s overall PA levels [58, 59]. A multitude of intervention types have been tested (e.g., policy, environmental, educational, coaching, and multi-component), making it difficult to conclude which strategies are most effective for improving children’s PA. A recent review suggests the importance of conducting community-based interventions that provide additional opportunities for PA within one’s community [13]. Some studies have used this approach to promote PA in children and youth [60–63]; however, these studies have failed to isolate the impact that providing access has on overall PA. In most of these studies, increased access is only one component of a multi-component intervention, making it difficult to identify whether this strategy *alone* can increase PA [64, 65]. Community-based interventions targeting PA are attractive for their potential to influence entire populations [57], including those most at risk for inactivity [66, 67]. Research has shown that such interventions are most effective and sustainable when involving collaboration among multiple sectors of the community [68], such as policy-makers, schools, service providers, practitioners, and academics.

**Fig. 1:**
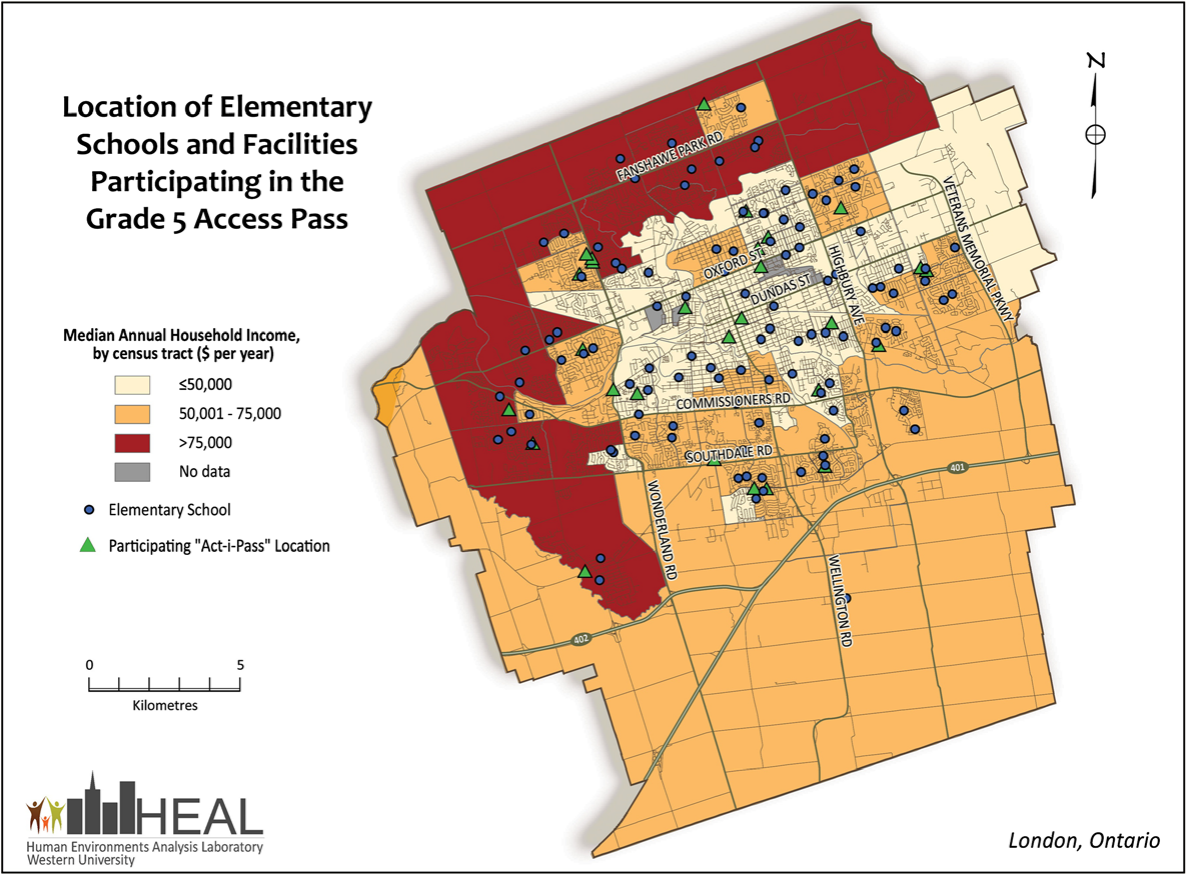
Location of elementary schools and facilities participating in the Grade 5 ACT-i-Pass program

The purpose of this study is to assess how the “Grade 5 Act-i-Pass” program – a naturally-occurring community-level intervention offering free access to PA opportunities – can lead to increased PA among children. The Grade 5 ACT-i-Pass program offers the entire population of grade 5 children in London, Canada free access to various PA facilities (e.g., arenas, recreation centres, pools) and programs (e.g., cardio-funk, skating, hip hop, basketball, floor hockey, swimming) well-distributed across the city for an entire school year (see Fig. 1 for locations). The pilot program has run for two years (2013–14, 2014–15). To our knowledge, no published study exists which has tested whether providing free access to PA facilities and programs *alone* will lead to increases in children’s PA levels during and after the intervention.

This study takes advantage of a novel, time-sensitive, population health research opportunity by using a two-year longitudinal cohort study design with validated mixed-methods data collection and analyses to assess potential change in PA among children given the ACT-i-Pass in 2014–2015. Thus, this study will fill a gap in the literature by evaluating the impact of the Grade 5 Act-i-Pass, an intervention offering free access to PA opportunities (facilities and programs), on grade 5 children’s PA level. The biggest hurdle to undertaking natural experiments is being able to identify an intervention in enough time to adequately design, fund, and conduct the ‘pre-test’ or ‘before’ stage of the intervention. The exceptional knowledge on the details (i.e., what, when, where) of a population-based PA intervention to be delivered to every grade 5 child attending school within London in 2014–15 provides us this unique opportunity.

## Methods & design

### Details of the intervention

The intervention being examined is a city-wide initiative being launched by London’s Child & Youth Network (CYN), called the *Grade 5 ACT-i-Pass* (ACT-i-Pass), which provides a free recreational access pass to all grade 5 children attending schools within the city limits. The ACT-i-Pass is designed to reduce financial barriers and increase the knowledge that children and their parents have about PA opportunities within London. The overarching goal of the ACT-i-Pass is to increase children’s PA levels.

**Fig. 2:**
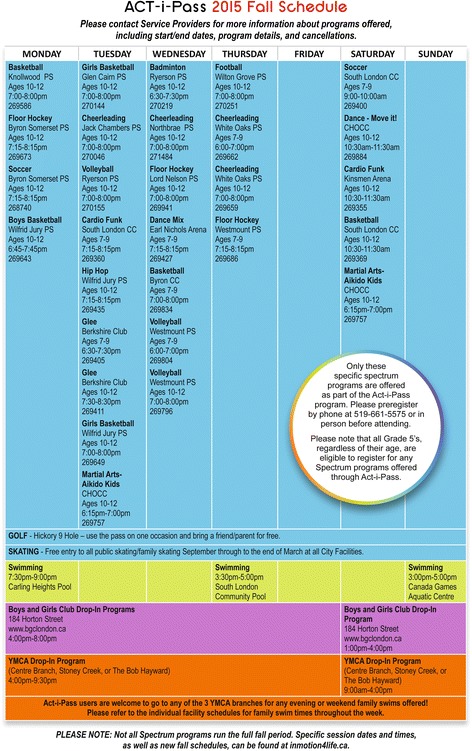
Sample ACT-i-Pass schedule from Fall 2015

In May 2014, all children in grade 4 were given an application package by a CYN rep at their school, including an information sheet clearly outlining details of the ACT-i-Pass and a parental consent form. Children were asked to return the signed consent form to the school representative by the end of May to provide the opportunity to collect baseline data. In September 2014, the children who returned a consent form were given an ACT-i-Pass. Passes were valid for the duration of the school year (Sept-June). The ACT-i-Pass grants each child “plus one” (e.g., friend, family member, or chaperone) free admittance to a variety of recreational programs at local facilities. A schedule (see Fig. 2 for an example) outlining the location, program, days, and times of each opportunity was given to the children, posted on a website (playeveryday.ca), and updated seasonally by CYN. Community partners include, but are not limited to: the YMCA of London, the Boys & Girls Club of London, and the City of London’s Parks and Recreation Department. Examples of programs include: soccer, skating, cardio funk, basketball, hip hop dance, skipping, volleyball, floor hockey, cheerleading, and open swims. These programs are offered outside of school-time, as after-school and weekend opportunities.

### Research question, objectives and hypotheses

Our overarching **research question** is: Does the provision of a free recreation access pass impact grade 5 children’s PA level? More specifically, we aim to better establish the potential causal effects of improving **access** to PA facilities and programs on children’s PA, in order to identify and disseminate information about effective intervention strategies. To address our research question, we propose three **specific objectives**.

#### Objective #1

**Fig. 3:**
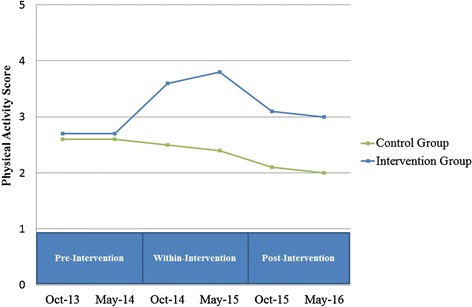
Hypothesized results for Objective #1, which states “we **hypothesize** that PA levels will be higher among children in the intervention group compared to children in the control group (control group is described below) at the 4 data collection points”

To assess **whether** the provision of the ACT-i-Pass increases children’s PA level during and after the intervention compared to a control group. We **hypothesize** that PA levels will be higher among children in the intervention group compared to children in the control group (control group is described below) at the 4 data collection points. Figure 3 shows how we hypothesize the intervention group will differ from the control group.

#### Objective #2

**Fig. 4:**
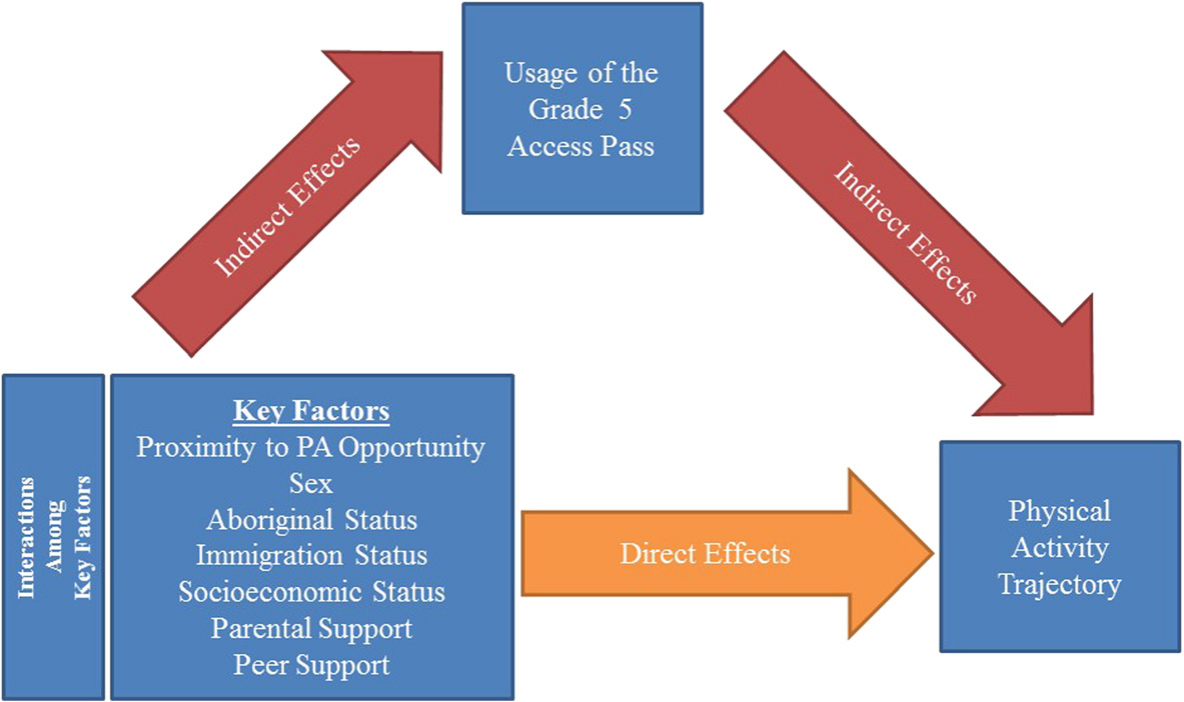
Conceptual model of how ACT-i-Pass changes the physical activity level trajectory among grade 5 children

To assess **how** and **why** child-specific trajectories of PA (between-children differences in PA level measured across time) in the intervention group differ according to children’s individual and neighbourhood characteristics. In light of previous studies, we have developed a **conceptual model** (Fig. 4). The model suggests seven key factors that are hypothesized to affect the level of usage of the ACT-i-Pass, and consequently, PA level: sex, socio-economic status, immigrant status, Aboriginal status, parental support, peer support, and proximity to a recreation facility (direct and indirect effects). In this model, the level of pass usage is positioned as a mediating variable – it is expected to directly affect the level of PA and, at the same time, it is affected by the seven factors.

**Fig. 5:**
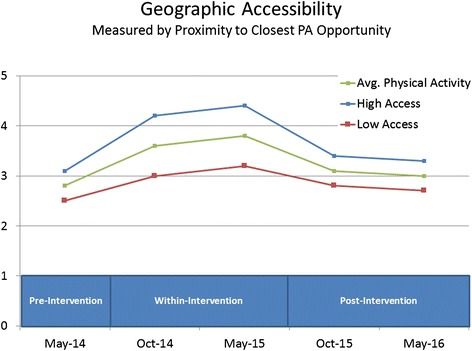
An example of hypothesized results for Objective #2, which states “we **hypothesize** that the intervention will have the most positive impact on PA levels among children who have high geographic access to ACT-i-Pass facilities”

Based on our literature review and conceptual model, we **hypothesize** that the intervention will have the most positive impact on PA levels among children who are: (a) frequent pass users; (b) female; (c) new immigrants to Canada; (d) Aboriginal; (e) from low socio-economic status neighbourhoods; (f) from households with supportive parent(s); (g) supported by peer(s); and (h) living in close proximity to one or more participating recreation facilities. We also expect interactions among factors. For instance, we hypothesize that the intervention will have a stronger effect among frequent pass users from low socio-economic status households compared to other frequent pass users. Figure 5 shows an example of how we hypothesize PA trajectories for a specific sub-group will differ from the trajectory of the intervention group average.

#### Objective #3

To **explore** additional factors that are unaccounted for in the conceptual model in order to gain a further understanding of why the ACT-i-Pass intervention may have had varying effects on changing PA levels.

### Study population and recruitment

The ***intervention group*** is the population of children who were in grade 4 in a London school as of May 2014. Grade 4 children were chosen as the study population as they provide an opportunity to evaluate how the ACT-i-Pass affects PA levels pre-intervention, within-intervention, and post-intervention through a longitudinal study design. Specifically, the same children will be surveyed once in grade 4 (pre-intervention: May 2014), twice in grade 5 (during the intervention: Oct 2014 and May 2015), and once again in grade 6 (post-intervention: Oct 2015).

Children in grade 4 were recruited from every public elementary school (*n* = 99) within the city limits. Within the 99 schools scheduled to distribute the ACT-i-Pass in May 2014, there are currently 3677 grade 4 children who were invited to participate. Based on previous experience [69–74], we expect 75 % of eligible children to participate (2757) and an attrition rate of approximately 8 % per year due to moving or loss of interest.

#### Recruitment

The protocol was approved by Western University’s Non-Medical Research Ethics Board (File#103954) and all 4 local school boards (2 English and 2 French boards). School principals were contacted about this project and information packages were sent home to inform parents that their children were eligible to participate. All material was available in both English and French. The packages included information about the ACT-i-Pass, the research project, and a parental consent form. After the children returned signed parent consent forms and child assent forms, they were enrolled and able to withdraw at any time. All children/parents in the target grade who receive a ACT-i-Pass but opt not to participate in the full study will be asked to complete a registration form, which we will use to control for selection bias. No study incentives were offered for participating in this part of the study.

To assess the effect of the intervention (Objective #1) a ***control group*** is needed. A non-equivalent control group of children in grades 5 and 6 will be used to compare to our within-intervention and post-intervention measurements respectively. Ideally, the control group would be comprised of randomly selected children of the same grade from London who do not receive the ACT-i-Pass; however, this is impossible as the population-based intervention provides an ACT-i-Pass to **all** grade 5 children. Thus, we use a non-equivalent control group of 566 grade 5children from London. PA levels among this group were assessed using the same measurement tools as used in the current study. We do acknowledge the data will be temporally mismatched, but are confident that this is the most equivalent control group that is completely unbiased by the provision of the ACT-i-Pass or any other community-level PA interventions. We expect that children from the intervention group and control group represent the same population of children (minimal period effect). We have made every effort to track and account for any time confounders that exist in this control group.

### Data collection

Data collection involves the use of a ***youth survey*** to address Objectives #1 and #2; a ***parent survey*** and ***ACT-i-Pass tracking*** to address Objective #2; and ***youth focus groups*** to address Objective #3.

#### Youth survey

The youth survey is a self-report survey that will be completed 4 times by the children over 3-years. The surveys will be completed in Spring 2014, Fall 2014, Spring 2015 and Fall 2015 to minimize differences in meteorological factors between measurement periods. Additionally, climate data from Environment Canada will allow us to control for weather in our statistical models. The survey will be administered in schools. Questions elicit information on socio-demographics (e.g., sex, race, family composition), postal code, sedentary behaviours, PA behaviours, barriers to PA, perceived accessibility to recreational facilities in their neighbourhood, and use of recreational facilities and programs. This tool was developed using previously-validated tools for a previous project (steamproject.ca), and has since been used successfully with over 1700 children in grades 4–8 throughout Southwestern Ontario. These questions are derived from the Physical Activity Questionnaire for Children (PAQ-C), which is a scientifically validated self-administered 7-day recall questionnaire that has high validity for reliably measuring general levels of PA in elementary school-aged children [75].

#### Parent survey

The online parent survey will be completed three times – pre-intervention (May 2014), within-intervention (May 2015), and post-intervention (October 2016) – for each project participant. This survey elicits additional information about household characteristics that children may not know and helps evaluate parental influences on PA. Household characteristics include parental education level, income, and current employment status. Parents were also asked to record their child’s height and weight and respond to items regarding perceptions of barriers to PA, recreational facility accessibility/use, and pass utility. This survey was developed and well-tested with parents participating in our previous research. As parental PA behaviour may mediate children’s PA (REF), we will also directly assess parental PA levels using the short version of the International Physical Activity Questionnaire (IPAQ).

#### ACT-i-Pass tracking

**Tracking of ACT-i-Pass** usage will be done by our community collaborators, the CYN partner organizations – the **City of London**, **YMCA**, and **Boys & Girls Club**. Each child is given a uniquely numbered pass. When a child uses the pass to access a program, the date, time, program name, facility location, and pass ID number are recorded and stored in an electronic database. At the end of each month of the ACT-i-Pass program, our team is provided with a secure digital copy of the complete database including detailed individual records of pass use throughout the month for each child.

#### Youth focus groups

Twelve semi-structured focus groups, each hosting six to ten child participants, will be held to elaborate on some of the findings and clarify potential gaps. Focus groups will be held in 12 schools after the end of the intervention (Winter 2015); schools will be selected to ensure diversity of socio-economic status (i.e., high, medium, low income) and geographic accessibility of PA opportunities (i.e., high/low number of ACT-i-Pass programs within walking distance [1.6 km] of the school). Focus groups will last 1–1.5 h and a semi-structured interview guide will be followed. Children will be asked to describe factors that facilitate/hinder their participation in PA in general and ACT-i-Pass in particular. Children will also brainstorm potential changes they would like to see (e.g., with respect to the ACT-i-Pass program, neighbourhood PA opportunities) to increase their willingness to participate in PA. Focus groups will be audio recorded and transcribed verbatim.

#### Parent interviews

Parent phone interviews will be conducted with the parents of the participants after the end of the intervention (Winter 2015); parents will be selected to ensure diversity of socio-economic status and geographic accessibility of PA opportunities. Interviews will last 15 to 30 min and a semi-structured interview guide will be followed. Parents will be asked about similar issues as the children, such as the factors that facilitate/hinder the participation and changes they would like to see in the ACT-i-Pass program. Interviews will be audio recorded and transcribed verbatim.

#### Service provider & school board focus groups

Six semi-structured focus groups will be held with ACT-i-Pass service providers and an additional six semi-structured focus groups will be held with representatives from the local school boards. Each focus group will last 45 to 60 min and host 4 to 8 participants. All ACT-i-Pass service providers and school board officials who were directly involved in the ACT-i-Pass program will be invited to participate, with additional focus groups added as necessary. These participants will be asked about the benefits of ACT-i-Pass, the sustainability of the program, and help to develop ideas that can improve the implementation of the program in the future. Focus groups will be audio recorded and transcribed verbatim.

### Measures

#### Youth physical activity levels

Youth PA is measured using the PAQ-C [75]. While the PAQ-C does not have the ability to provide estimates of PA intensity, it has consistently high validity for reliably measuring general levels of PA in elementary school-aged children [75]. The questions in the PAQ-C are designed to utilize memory cues, such as lunch and evening items, to enable easier recall of children’s PA levels [75]. The PAQ-C includes 10 items and asks individuals to rate how much PA they have done over the past week [75]. Based on each of the item responses, an overall mean score ranging from 1 to 5 is calculated, where a higher score indicates a higher PA level. A score will be calculated for each child for each of the 5 youth survey periods. This score will be used to establish if the children’s PA levels increased during and post-intervention.

#### ACT-i-Pass usage

ACT-i-Pass usage will be used as an explanatory variable to highlight change in PA. The key hypothesis in Objective #2 states that children who use the pass more often will also experience more positive changes on PA levels than children who do not use the pass. This variable will allow us to compare whether children with higher pass use have greater increases in PA levels over time than those who do not use the pass (or use it less often). The variable will be provided as a continuous count of the number of times a child has used (i.e., swiped) the pass over the course of the intervention.

#### PA program and facility use

The ACT-i-Pass tracking will also provide objective measures of which PA programs and facilities are used by each pass holder during the intervention. By knowing which facilities and programs are used more/less often by children from different neighbourhoods and schools in advance of the youth focus groups, we will be able to better form and direct focus group questions to meet Objective #3.

#### Parental physical activity

A measure of parents PA behaviour is derived from their responses to the short version of the IPAQ. The IPAQ will provide information on time spent sitting, walking, in moderate intensity PA, and in vigorous-intensity PA. Following the official IPAQ scoring protocol (http://www.ipaq.ki.se), the total daily metabolic equivalent scores are calculated by summing the product of reported time within each item by a metabolic equivalent value specific to each category of PA.

#### Parental support

Two measures of parental support are derived from a series of questions on both the Youth Survey ***and*** the Parent Survey. Questions focus on 4 elements of parental support that have been linked to children’s PA: encouraging children to do PA, providing transportation to places to do physical activities or sports, watching children participate, and actively playing with children [76, 77]. In both surveys, the questions are posed to elicit responses about how often during a typical week, on a 5-point Likert scale: (0) Never, (1) 1–2 days, (2) 3–4 days, (3) 5–6 days, (4) every day. The responses from each child for the 4 questions will be averaged and combined for a *child perception of parent support* score. The same procedure will be completed with parent survey responses to generate a combined score for *perception of parental support*.

#### Peer support

Measures of peer support for PA are derived from a set of 4 questions on the Youth Survey. The questions focus on the presence of encouragement from friends, PA behaviour of friends, and peer victimization (e.g., teasing from friends or classmates). These questions are posed to elicit responses about how often during a typical week, on a 5-point Likert scale: (0) Never, (1) 1–2 days, (2) 3–4 days, (3) 5–6 days, (4) every day. The responses from each child for the 4 questions will be averaged and a combined peer support score calculated for incorporating into statistical models as potential mediating factors of children’s PA.

#### Socio-demographic factors

Sex, immigrant status, Aboriginal status, and ethnicity will be determined from items on the youth survey. Additional household characteristics such as parental education level, income, and current employment status will be determined from questions on the parent survey. Socio-economic status will be calculated using the “social distress index” following procedures previously used by Gilliland [70] with the 2011 Census data at the dissemination area level (the smallest geographic unit for which socioeconomic data are available).

#### Proximity to the nearest PA opportunity

Proximity will be measured objectively in GIS (ArcGIS 10.1) as the shortest distance (along the street network) in two ways: between a child’s home postal code and the nearest public recreation facility, and between a child’s school and the nearest public recreation facility. Additionally, two youth survey items ask about the children’s knowledge and perceived access to recreation facilities in their neighbourhood.

### Data analysis

#### Objective #1

To assess whether or not the provision of the ACT-i-Pass increases the level of PA during the intervention and whether these effects are sustained beyond the period of the intervention and to test the hypotheses, we will employ a quasi-experimental design with non-equivalent control group [78]. This design is considered one of the most reliable techniques to measure the effects of population-based interventions. Evaluation of quasi-experimental studies is relatively straightforward as the outcomes of subjects from the intervention group can be compared to those from the control group. This analysis is commonly referred to as an intent-to-treat analysis (ITT); its results are unequivocal and easy to explain to policy analysts, government officials, and the public.

However, population-based interventions are affected by a number of complications. Non-compliance occurs when subjects in the intervention group refuse to participate. It is expected that some parents/guardians will not permit their children to participate in the ACT-i-Pass intervention when it is implemented. If all subjects in the intervention group do not participate in an intervention, the ITT analysis confounds two distinct phenomena: program efficacy (program impact among those who actively participate) and program uptake (compliance with program activities) [79]. Little and Yau [80] argue that in the presence of non-compliance, a conventional ITT analysis estimates the causal effect of group allocation (intention to treat or encouragement) rather than the effect of the treatment actually received. From a policy point of view, it is important to assess both the overall effect of the ACT-i-Pass on all children from the intervention group and the program impact on children who will participate in the ACT-i-Pass. The results obtained through each of these two assessments, when combined together, can better inform policymakers about the effect of the ACT-i-Pass on children’s PA. To estimate the effect of participation in (receipt of) ACT-i-Pass, we will employ the Complier Average Causal Effect (CACE) estimation approach [80–83], which is sometimes referred to as the “Local Average Treatment Effect” [84].

#### Effect definition

We defined the ITT effect as a series of differences between the average PA level for children from the intervention group and the average PA level for children from the control group at three data collection points (October and May in grade 5 and October in grade 6), regardless of whether children assigned to the intervention group participate in the ACT-i-Pass. We defined the CACE effect as the difference between the average level of PA for children from the intervention group who participated in the ACT-i-Pass and the average level of PA for children from the control group who would participate in the ACT-i-Pass, if the intervention were offered to them.

#### Estimation

The ITT and CACE effects will be analyzed through structural equation modelling (SEM) techniques [85]. To estimate the unknown compliance status of children in the control group and to estimate average intervention effects for compliers, we will employ the maximum-likelihood estimation method using the expectation-maximization algorithm. The theoretical and practical aspects of this method are presented in detail by Jo and Muthén [86] and by Muthén and Muthén [87]. The ITT and CACE analyses will be carried out using the Mplus program [87] which offers superior handling of missing data due to attrition and non-compliance (Full Information Maximum Likelihood algorithm).

#### Objective #2

The second objective of this study is to assess whether the across-time changes in PA scores are a function of the proposed child- and neighbourhood-level factors. To test hypotheses related to this objective, we will use various latent growth curve modeling (LGC) techniques within the context of structural equation modeling [88–91]. First, we will assess the overall PA trajectory for all children from the intervention group across 4 measurement points. Second, we will test if (and how) each of the proposed factors affects the shape of the PA trajectory. Results from these models will allow us to find out how much each of the proposed factors affect PA trajectory, controlling for other covariates. Figure 4 presents our proposed conceptual model. It posits that PA is a time-dependent process, rather than a static characteristic. PA levels will be observed from May 2014 (grade 4) until May 2016 (grade 6). We also propose that the effects of some predictors (pass usage, socio-economic status, proximity) on PA trajectories might vary at different time points during the intervention. Understanding the dynamics of these changing relationships may be crucial to the design of efficient population-level PA interventions. The background variables (age, ethnicity, BMI, etc.) will be considered in all model analyses, to adjust for missing data and selection bias.

#### Objective #3

The third objective is to explore additional factors that are unaccounted for in the proposed conceptual model to gain a further understanding of why the ACT-i-Pass had varying effects on changing PA levels. This objective will be achieved using the qualitative data collected via focus groups. Data collection and analysis will take place simultaneously using a combination of the editing and template organizing styles outlined by Miller and Crabtree [92]. A minimum of two team members will independently conduct inductive content analysis on each transcript and compare their findings. NVivo software will be utilized to code and categorize emerging themes. A number of strategies will be employed to ensure the trustworthiness of the findings, such as member-checking, peer debriefing, and using multiple coders [93]. The qualitative data from the focus groups will provide extensive information on additional factors that are unaccounted for in the proposed conceptual model to provide a fuller understanding of why the ACT-i-Pass had varying effects on changing PA levels across the intervention population. This breadth and depth of understanding will be critical to the future development of PA interventions.

## Discussion

Innovative research projects are necessary to help identify what strategies are effective for increasing children’s PA at the population level to ultimately improve the overall health of Canadian children. Innovations in this interdisciplinary collaboration will arise from combining expertise from the fields of geography, epidemiology and biostatistics, paediatrics, kinesiology, sociology, and health promotion, with the decision-making powers and practical experience associated with local policymakers, health professionals, community organizations, and recreation service providers. This project will build research capacity in the area of children’s health through the exchange of knowledge across sectors (in a true university-community collaboration).

This study proposes a novel longitudinal approach to comprehensively evaluate the effectiveness of a community-driven population health intervention to increase children’s PA levels. To our knowledge, no published studies exist which have tested whether or not providing free access to PA facilities and programs alone will lead to increases in children’s PA levels during and after the intervention. The results of this study can inform policy- and decision-makers about the sub-groups of the population that benefitted the most (or least) from the intervention to provide more specific information on how to develop and target future interventions. If the program is successful, local community organizations and policymakers at all levels of government (municipal, provincial, and federal) may consider adopting the program more widely across Canada, thereby having a larger impact on the health of Canadian children.

## Trial status

This study is funded by the Canadian Institutes of Health Research (CIHR-IPPH) and the Canadian Cancer Society. The study has received Research Ethics Board approval by University of Western Ontario (REB#10394) and the four school boards and one private school in London, Canada. The research team is currently conducting the third round of surveys that are part of the recruitment and data collection process and are progressing as envisioned in this protocol.

## Abbreviations

ACT-i-Pass: The Grade 5 ACT-i-Pass is an initiative that gives grade 5 students in the city of London, Canada the opportunity to receive free access to recreation programs and facilities within the city. More details about the project can be found at the project websites: http://playeveryday.ca and http://inmotion4life.ca/actipass.
CACE: The complier average causal effect estimation approach compares the difference between the average outcomes of the intervention group compared with the average outcomes of the control group.
CYN: The Child & Youth Network (londoncyn.ca) is made up of over 170 agencies and individuals in London, Canada from the education, health, recreation and social services sectors. The CYN is dedicated to helping build strong families and breaking down the barriers that put our children, youth and families at risk. They do this by collectively planning, facilitating collaboration, building awareness, providing education and improving access to services.
IPAQ: The International Physical Activity Questionnaire is a widely-used, previously-validated, tool that has reasonable measurement properties for monitoring population levels of PA.
ITT: Intent-to-treat is a type of analysis that allows outcomes of subjects from an intervention group to be compared to those from a control group.
PA: Physical activity refers to the movement of one’s skeletal muscles that require energy expenditure.
PAQ-C: The Physical Activity Questionnaire for Children (PAQ-C) is a scientifically-validated self-administered 7-day recall questionnaire that records children’s self-reported physical activity levels.
SEM: Structural equation modelling is a technique for simultaneously estimating the relationships between observed and latent variables
